# The Ability of Riboflavin-Overproducing *Lactiplantibacillus plantarum* Strains to Survive Under Gastrointestinal Conditions

**DOI:** 10.3389/fmicb.2020.591945

**Published:** 2020-10-22

**Authors:** Annel M. Hernández-Alcántara, Sandra Pardo, Mari Luz Mohedano, Graciela M. Vignolo, Alejandra de Moreno de LeBlanc, Jean Guy LeBlanc, Rosa Aznar, Paloma López

**Affiliations:** ^1^Department of Microorganisms and Plant Biotechnology, Margarita Salas Center for Biological Research (CIB)-Consejo Superior de Investigaciones Científicas (CSIC), Madrid, Spain; ^2^Reference Center for Lactobacilli (CERELA)-Consejo Nacional de Investigaciones Científicas y Técnicas (CONICET), San Miguel de Tucumán, Argentina; ^3^Department of Preservation and Food Safety Technologies, Institute of Agrochemistry and Food Technology (IATA)-Consejo Superior de Investigaciones Científicas (CSIC), Paterna, Spain; ^4^Department of Microbiology and Ecology, University of Valencia, Valencia, Spain

**Keywords:** lactic acid bacteria (LAB), riboflavin, protein mCherry, *Lactiplantibacillus plantarum*, tolerance to gastrointestinal stress of *Lactiplantibacillus*

## Abstract

Riboflavin, vitamin B2, is essential for humans and has to be obtained from the diet. Some lactic acid bacteria (LAB) produce this vitamin, and they can be used for *in-situ* fortification of foods. This could be an alternative to supplementation with chemically synthesized vitamin, to palliate riboflavin deficiencies in specific groups of people. Moreover, if the producing LAB could survive in the gastrointestinal stress (GIT) they could be added as probiotics in this environment. In the present study we tested two riboflavin-overproducing *Lactiplantibacillus plantarum* strains (M5MA1-B2 and M9MG6-B2), spontaneous mutants of LAB isolated from chicha, a traditional Andean beverage. These two LAB, and also their isogenic strains M5MA1-B2[pRCR12] and M9MG6-B2[pRCR12], expressing the mCherry protein from the pRCR12 plasmid, were evaluated *in vitro* under simulated GIT conditions. Among other, specifically developed protein fluorescence assays were used. The four LAB showed similar levels of adhesion (>6.0%) to Caco-2 cells, higher than that of the probiotic *Lacticaseibacillus rhamnosus* GG strain (4.51%). Thus, LAB biofilm formation was assessed in the labeled cells by intracellular mCherry fluorescence and in the unlabeled parental strains by crystal violet staining. Both methods detected the formation of consistent biofilms by the *L. plantarum* strains. The quantification of mCherry fluorescence was also used to analyze LAB auto-aggregation properties. High levels of auto-aggregation were detected for both M5MA1-B2[pRCR12] and M9MG6-B2[pRCR12]. Survival of LAB included in a commercial cereal-based food matrix (Incaparina) under GIT conditions was also evaluated. The four LAB were resistant *in vitro* to the stomach and intestinal stresses, and proliferated in this environment, indicating a protective and nutritional effect of the Incaparina on the bacteria. Also, M9MG6-B2 survival in the presence or absence of Incaparina was evaluated *in vivo* in a BALB/c mouse model. The administration of the M9MG6-B2 strain alone or together with Incaparina had no adverse effect on the health, growth and/or well-being of the rodents. In addition, an increment in the villus length/crypt depth ratio was observed. The overall results obtained indicate that the LAB studied have probiotic characteristics of interest for the development of functional foods.

## Introduction

Riboflavin (vitamin B2) is essential for life, but unlike many plants, fungi, and bacteria, humans are unable to synthesize it. Humans must therefore rely on exogenous sources of riboflavin provided in their diet, and also by its production by the microbiota of the large intestine (Hill, 1997; Powers, 2003). Important food sources of riboflavin included milk and dairy products, yeast, cereals, meats, oily fish, and green leafy vegetables. Generally grain products provide low amounts of riboflavin as much of the vitamin is lost due to processing; nevertheless, vitamin fortification practices make certain breads and other grain-based products good sources of riboflavin (Powers, 2003).

Riboflavin is very important for energy production, since it is the precursor of the two flavoprotein coenzymes, flavin adenine dinucleotide (FAD) and flavin mononucleotide (FMN). These coenzymes are crucial in many cellular process as they are cofactors in oxidation-reduction reactions; conversion and recycling of niacin, folate and vitamin B6, the synthesis of all heme proteins including hemoglobin, nitric oxide synthases, P450 enzymes, and in electron transfer and oxygen transport and storage (Pinto and Rivlin, 2013). These flavoproteins are also co-factors in the metabolism of essential fatty acids in brain lipids, the absorption and utilization of iron, and the regulation of thyroid hormones. Therefore, the interruption of any of these processes due to a riboflavin deficiency would have negative consequences for many body functions including brain functions (Kennedy, 2016). Riboflavin derivatives also have antioxidant properties and increase endogenous antioxidant status as essential cofactors in the glutathione redox cycle (Ashoori and Saedisomeolia, 2014).

The World Health Organization (WHO) has proposed riboflavin, as one of the six major indicators for assessing human growth, development, and nutritional status, and it was reported that riboflavin deficiency is endemic in populations whose diets lack dairy products and meats (Rohner et al., 2007). The European Food Information Council (EFSA, 2017), has established that the recommended daily allowance (RDA) of riboflavin is 1.7 mg/day; however, this can only be obtained by consuming balanced diet. The increased incidence of malnutrition, in addition to certain pathologies and drugs that affect absorption, is why sub-clinical deficiencies of riboflavin are very frequent throughout the world. For this reason, mandatory fortification of staple foods is now recommended as a general health policy in many countries. Currently, industrial production of riboflavin is mainly via microbial biosynthesis, and among the producer strains are two yeast-like fungi *Eremothecium ashbyii* and *Ashbya gossypii*, as well as the bacterium *Bacillus subtilis* (Alizadeh Behbahani et al., 2019).

Some lactic acid bacteria (LAB) are also able to synthesize B-group vitamins and have potential for their *in situ* production in fermented foods (Capozzi et al., 2011; Juarez del Valle et al., 2014). In this way, the use of riboflavin producing LAB to produce novel bio-enriched foods, and that also provide other health benefits, represents a more natural and consumer-acceptable alternative to using chemically synthesized vitamins (Gu and Li, 2016; Rodrigo-Torres et al., 2019). The levels of the vitamin that would be produced in such foods presumably would not have any negative health implications for humans. No upper limit of intake has been set for riboflavin in humans since excess intakes are excreted in urine (Flynn et al., 2003). In this regard, Institute of Medicine Food Nutrition Board (1998) and the European Food Safety Authority (2000) have both stated that intakes of up to 400 mg of riboflavina per day, almost 400 times the recommended intake, did not cause adverse side effects and concluded that this value could be used as the Tolerable Upper Intake Level.

Roseoflavin is a toxic riboflavin analog, and can be used to select mutations in the riboswitch regulatory region of the riboflavin (*rib*) operon, which results in constitutive expression of the riboflavin operon. This in turn leads to over-production of vitamin B2 (Kukanova et al., 1982). Hence, roseoflavin treatment is a widely used methods to obtain LAB strains that overproduce B2 vitamin (Capozzi et al., 2011; Juarez del Valle et al., 2014). This method has been successfully employed for *Limosilactobacillus fermentum, Lactiplantibacillus plantarum, Lactococcus lactis, Leuconostoc mesenteroides*, and *Propionibacterium freudenreichii* (Burgess et al., 2004, 2006; Arena et al., 2014), among others. In particular, a dairy product fermented with *P. freudenreichii* was shown to counteract the deficiency of riboflavin in an animal model (LeBlanc et al., 2006). An important characteristic to highlight, is that these roseoflavin resistant strains, are spontaneous, non-genetically modified, organisms and therefore could be exploited in the production of foods enriched with vitamin B2.

The concept of *in situ* production of riboflavin using selected LAB opens the possibility of developing novel food products designed for specific groups of people, such as the elderly, children, pregnant women, sportsmen, vegetarians, and adolescents (Ge et al., 2020), and even to use the food to deliver probiotic riboflavin-overproducing LAB for *in situ* vitamin B2 synthesis in the digestive tract. The chemical structure of riboflavin produced by LAB is the same as that used to supplement foods, but its production costs are much lower (Liu et al., 2020). In addition, probiotic strains could provide the host not only with specific health promoting properties, but could also increase *in situ* production of riboflavin (Thakur et al., 2016). It has even been shown that riboflavin producing probiotic strains can provide anti-inflammatory and anti-cancer effects in animal models (LeBlanc et al., 2020), but the safety of using live vitamin-producing probiotics needs to be examined further before being administered to immuno-compromised patients.

In this context, *L. plantarum* strains isolated from chicha (a traditional beverage from Andean regions), were exposed to roseoflavin, and riboflavin-overproducing strains were isolated and tested for the production of functional cereal-based foods, with the objective that the technological properties they could provide in this type of food could palliate the deficiency of this vitamin in humans (Yépez et al., 2019).

These vitamin B2-overproducing LAB have also been used to standardize fluorescent detection of riboflavin production in real time during growth (Mohedano et al., 2019) and also showed the potential of strains *L. plantarum* M5MA1-B2 and M9MG6-B2, as producing high levels of riboflavin and possessing good technological properties. Both strains carry a different punctual mutation in the same position (G19A and G19C in the M5MA1-B2 and M9MG6-B2 strains, respectively) located in the regulatory region of the *rib* operon (Mohedano et al., 2019). Furthermore, the comparative analysis of the genomes of five of the parental riboflavin producing strains, as well as assessing some criteria to determine their food safety, revealed a great similarity between the strains studied. The M5MA1 strain was also shown to possess some unique genes in its genome (Rodrigo-Torres et al., 2019).

Therefore, the objective of this work was to evaluate some probiotic properties of the *L. plantarum* M5MA1-B2 and M9MG6-B2 strains using *in vitro* comparative analysis of behavior and survival in the human digestive tract followed by an *in vivo* evaluation of M9MG6-B2 in a cereal-based food matrix using a murine model.

## Materials and Methods

### Bacterial Strains and Growth Conditions

The LAB used in this work are presented in Table 1. The LAB strains were routinely grown in MRS broth (de Man et al., 1960) (Pronadisa, Spain) at 37°C. When evaluating the riboflavin production, the strains were grown in the chemical defined medium (CDM) (Sánchez et al., 2008) lacking riboflavin (CDM-Rib). LAB carrying the plasmid pRCR12 were growth in media supplemented with chloramphenicol (Cm) at 10 μg mL^−1^, besides in the experiments performed in microtitre plates, in which the antibiotic was omitted. Solid media were prepared by addition of agar at a concentration of 1.5% (w/v) (Pronadisa, Spain).

**Table 1:** Lactic acid bacteria used in this study.

**Strain**	**N° in type collections.**	**Plasmid**	**Antibiotic resistance**	**Characteristics**	**References**
*Lactobacillus plantarum* M9MG6-B2. Renamed *Lactiplantibacillus plantarum* M9MG6-B2	CECT 9435	–	–	Vitamin B2- overproducing strain	Yépez et al., 2019
*Lactobacillus plantarum* M5MA1-B2. Renamed by Zheng et al. (2020) *Lactiplantibacillus*	CECT 9434	–	–	Vitamin B2- overproducing strain	Yépez et al., 2019
*Lactiplantibacillus plantarum* M9MG6-B2 [pRCR12]	–	[pRCR12]	Cm^R^	M9MG6-B2 strain fluorescently labeled with mCherry	This study
*Lactobacillus plantarum* M5MA1-B2 [pRCR12]. Renamed by Zheng et al. (2020) *Lactiplantibacillus*	CECT 9402	[pRCR12]	Cm^R^	M5MA1-B2 strain fluorescently labeled with mCherry	Mohedano et al., 2019
*Lactococcus lactis* MG1363[pRCR12]	–	[pRCR12]	Cm^R^	Source of plasmid pRCR12	Garay-Novillo et al., 2019
*Lactobacillus rhamosus* GG. Renamed by Zheng et al. (2020) *Lacticaseibacillus*	ATCC 53103	–	–	probiotic strain	Capurso, 2019

*CECT, Colección Española de Cultivos Tipo; ATCC, American Type Culture Collection*.

### Fluorescent Labeling of *L. plantarum* M9MG6-B2

The pRCR12 plasmid was obtained from *L. lactis* MG1363[pRCR12], grown until the beginning of stationary phase, using the JetStar 2.0 Plasmid Purification kit (Genomed, Löhne, Germany). Plasmid isolation was performed following the protocol supplied with the kit. For the insertion of plasmid pRCR12 into *L. plantarum* M9MG6-B2, 0.5 μg of plasmid were used for electroporation (25 μF, 1.3 kV and 200 Ω in 0.1 cm cuvettes) as previously described (Berthier et al., 1996). Transformants were selected on MRS-agar supplemented with Cm at 10 μg mL^−1^ at 37°C.

### Simultaneous Detection of Bacteria Growth and Riboflavin or mCherry Production

To follow the growth and fluorescence of *L. plantarum* strains in real time, the method described by Mohedano et al. (2019) was used. Simultaneous spectrophotometric monitoring of growth at 37°C and of the fluorescence of the bacterial cultures was performed using the Varioskan Flask System (Thermo Fisher Scientific, USA) and sterile 96-well optical white w/lid cell culture polystyrene plates (Thermo Fisher Scientific, USA). The experiments were performed in triplicate by incubating at 37°C and measuring the optical density (OD) and fluorescence every 30 min during 14 h.

Growth and fluorescence due to mCherry protein were determined in cultures grown in MRS that were sedimented by centrifugation at 9,300 × *g*, for 10 min at room temperature and resuspended in fresh medium to an OD_600 nm_ of 0.1. Aliquots of 200 μL of each culture were analyzed in triplicate in the microtiter plate reader incubated at 37°C, using MRS as control. The OD_600 nm_ of the cultures was measured and the emission of the mCherry fluorescence at a wavelength of 610 nm upon excitation at a wavelength of 587 nm.

Growth and fluorescence due to riboflavin production were determined from cultures growth in MRS that were sedimented as above and resuspended in CDM-Rib to an initial OD_480 nm_ of 0.1. Aliquots of 200 μL of each culture were analyzed in triplicate as indicated above. Growth was monitored at an OD_480 nm_ and the riboflavin fluorescence upon excitation at a wavelength of 440 nm and detection of emission at a wavelength of 520 nm.

The growth of the LAB in liquid medium allowed the determination of the kinetic parameters, doubling time or generation time (dt) and growth rate (μ) as described by Maier (2009) and Widdel (2010).

### Quantification of Riboflavin Production by Fluorescence

To quantify the riboflavin concentration, a calibration curve was constructed to correlate the fluorescence emitted by solutions with increasing concentrations of riboflavin at 520 nm (Supplementary Figure 1). To this end, serial dilutions of a riboflavin solution prepared in CDM-Rib medium at a concentration of 10 mg mL^−1^ were analyzed by placing aliquots of 200 μL of each dilution in a black, non-sterile, polypropylene 96-well plate (Nunc™, Thermo Fisher, USA), at an excitation wavelength of 440 nm and an emission wavelength of 520 nm in the Varioskan plate reader.

Quantification of riboflavin levels produced by the LAB strains was performed by measuring fluorescence in culture supernatants grown in CDM-Rib medium using 14 h cultures described in section Simultaneous Detection of Bacteria Growth and Riboflavin or mCherry Production that were recovered from each well after bacteria were removed by centrifugation. Aliquots of 200 μL of the supernatants were used to measure fluorescence in the plate reader. Riboflavin concentration was estimated by interpolating fluorescence values on the calibration curve. The determinations were made in triplicate.

### Analysis of Bacterial Cultures by Fluorescence Microscopy

Exponential cultures of the *L. plantarum* strains were sedimented by centrifugation and resuspended in saline solution (0.85% w/v NaCl) to obtain a 10-fold concentrated suspension. Subsequently, a 5 μL aliquot of the suspensions was directly analyzed without fixation by phase contrast or fluorescence microscopy. A Leica DM1000 model microscope (Leica Microsystems, Mannheim, Germany) with a light source EL6000 and the filter system TX2 ET was used for detection of the mCherry fluorescence. The microscope was connected to a DFC3000G camera (Leica Microsystems) with a CCD sensor. Image analysis was performed with the Leica Application Suite X software (Leica Microsystems).

### Caco-2 Cell Culture and Adhesion Assay

The human enterocyte cell line, obtained from the cell bank at the Centro de Investigaciones Biológicas Margarita Salas (Madrid, Spain), was seeded in 96-well tissue culture plates (Falcon Microtest™, USA) at a final concentration of 1.25 × 10^5^ cells mL^−1^ and grown as monolayers of differentiated and polarized cells for 14 days as previously described (Nácher-Vázquez et al., 2017). For the adhesion assays, exponential-phase LAB cultures grown in MRS were sedimented by centrifugation (12,000 × *g*, 10 min, 4°C), and resuspended in the appropriate volume of Dulbecco's Modified Eagle medium (DMEM, Invitrogen) to give a final concentration of 1.25 × 10^6^ colony forming units (cfu) mL^−1^. Each bacterial suspension (0.1 mL) was added to a well (ratio 10:1, bacteria:Caco-2 cells) and the plates were incubated for 2 h at 37°C. The non-adhered bacteria were then removed and the cell-associated bacteria processed and quantified by plate counting on MRS-agar plates, as previously described (Nácher-Vázquez et al., 2017). All adhesion assays were conducted in triplicate. The percentage of adhesion to Caco-2 cells was calculated as:

$$\begin{array}{l} Adhesion\;\left(\%\right)=\frac{\frac{cfu}{mL}\;attached\;to\;Caco-2\;cells}{\frac{cfu}{mL}\;added\;to\;Caco-2\;cells}\times 100 \end{array}$$

### Detection of Biofilm Production on Polystyrene Plates as Abiotic Surface

#### Crystal Violet Staining Method

Assessment of biofilm formation on polystyrene plate was conducted as previously described by Stepanovic et al. (2007). LAB were grown in MRS medium until a concentration of 1.5 × 10^8^ cfu mL^−1^ was reached and then diluted 1:100 in the same medium. Then, 200 μL of these suspensions (1.0 × 10^6^ cfu mL^−1^) were used to inoculate sterile 96-well polystyrene microtiter plates (BD, New Jersey, USA). MRS medium, was used as negative control (Ctr). After incubation for 48 h at 37°C the supernatant from each well was carefully removed to eliminate the planktonic cells, then wells were gently washed 3 times with sterile PBS (Phosphate-Buffered Saline) and dried prior the addition of 150 μL of methanol, as fixing agent, and incubation for 20 min. Afterwards, methanol was removed from the wells and biofilms subsequently stained with 150 μL of crystal violet solution (2.0% w/v) (Fluka, USA) for 15 min at 21°C. The staining solution was then removed from the wells followed by two washes with distilled water to eliminate the excess of dye and dried. Finally, the dye was resolubilized with 150 μL of ethanol (96% v/v) for 30 min and the stained bacteria were quantified by measuring the OD_570 nm_ of the sample (OD_S_) in comparison with the OD_570 nm_ of the control sample (OD_Ctr_) in the Varioskan plate reader. Bacteria were classified for their ability to form biofilms in four categories according to Hashem et al. (2017): (i) non-adherent or negative biofilm, when sample OD_570 nm_ (OD_S_) ≤ negative control OD_570 nm_ (OD_Ctr_); (ii) weakly adherent, OD_Ctr_ < OD_S_ ≤ (2 x OD_Ctr_); (iii) moderately adherent, (2 x OD_Ctr_) < OD_S_ ≤ (4 x OD_Ctr_) and (iv) highly adherent, (4 x OD_Ctr_) < OD_S_. Three cultures of each bacterium were analyzed, and experiments were performed in triplicate.

#### Fluorometric Method by mCherry Fluorescence Measurement

A new direct quantitative method, developed in this work, and based on the fluorescence emitted by the mCherry protein encoded by pRCR12 was used. The LAB biofilm formation was carried out as described in section Crystal Violet Staining Method. After 48 h of incubation, the biofilms were carefully washed three times with 200 μL of PBS. Subsequently, each well was resuspended in 200 μL of saline solution and the fluorescence determined as the emission of the mCherry fluorescence at a wavelength of 610 nm upon excitation at a wavelength of 587 nm was measured. Results were expressed as arbitrary units (au) of fluorescence emitted by each *L. plantarum* strain.

### *In vitro* Auto-Aggregation Test

A method based on the detection of the mCherry fluorescence and developed in this work was used. A calibration curve for each *L. plantarum* strain was constructed to correlate the fluorescence emitted by the suspensions with the concentration of bacteria (Supplementary Figure 2). For this purpose, serial dilutions of a bacterial suspension prepared in saline solution at a concentration of 2.0 × 10^8^ cfu mL^−1^ were analyzed by placing aliquots of 200 μL of each dilution in a 96-well polystyrene plate to measure fluorescence due to mCherry at an excitation wavelength of 578 nm and an emission wavelength of 610 nm in the Varioskan plate reader. The results obtained showed a linearity range between 2.5 × 10^7^ cfu mL^−1^ and 2 × 10^8^ cfu mL^−1^.

To determine the auto-aggregation ability of *L. plantarum* strains, exponential cultures grown in MRS were sedimented and resuspended to a final concentration of 1.0 × 10^8^ cfu mL^−1^ in saline solution. Subsequently, 200 μL aliquots of the bacterial suspensions were dispensed into a 96-well polystyrene plate and fluorescence was measured for detection of mCherry in the Varioskan equipment, before (initial fluorescence) and after (final fluorescence) incubation at 37°C without shaking for 15 h. Non-aggregated bacteria were carefully removed and the aggregated cells at the bottom of each well were resuspended in 200 μL of saline solution prior to determine the final fluorescence. The auto-aggregation was calculated as percentage, being 100% the initial fluorescence:

$$\begin{array}{l} \%\;Auto-aggregation\;=\\ \frac{Initial\;fluorescence-Final\;fluorescence}{Initial\;fluorescence}\times 100 \end{array}$$

### Resistance to Simulated Gastrointestinal (GIT) Transit in a Food Matrix

**Figure 1:**
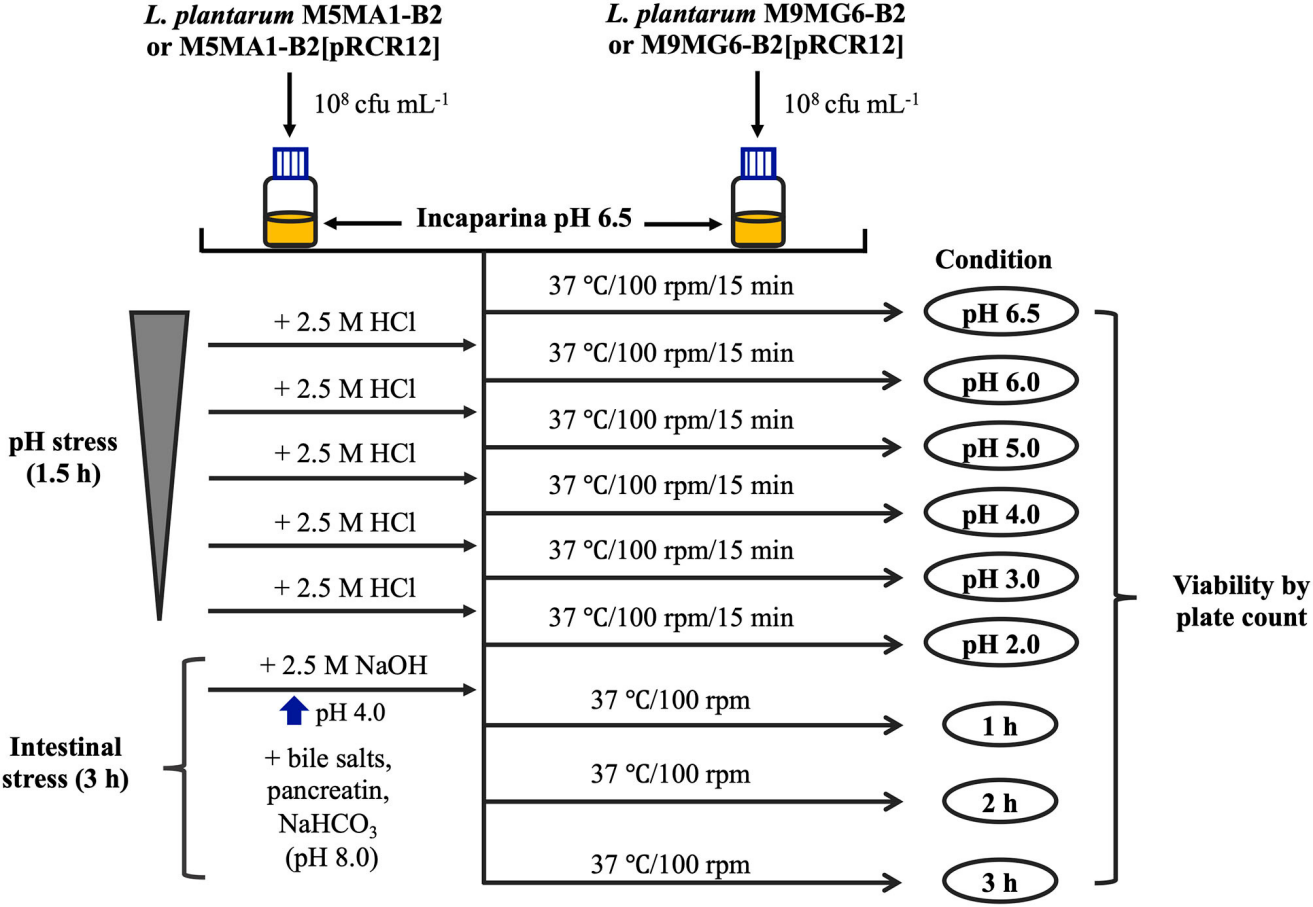
Experimental *in vitro* protocol for GIT stress. Aliquots of 30 mL of the food matrix, Incaparina (pH 6.5), prepared according to the instructions of the product, were inoculated with either M5MA1-B2, M5MA1-B2[pRCR12], M9MG6-B2, or M9MG6-B2[pRCR12] strains. Subsequently, suspensions were subjected to sequential increasing acidic stress, by gradually decreasing the pH from 6.5 to 2.0. After 15 min of incubation at each pH, aliquots were withdrawn to determine cell viability. After gastric treatment, pancreatin and bile salt juices were added to the gastric phase to simulate intestinal stress. The whole mixture was incubated for 3 h and after every hour cell viability was measured.

The food matrix used in the trials was the cereal-based beverage Incaparina (Central de Alimentos, S.A., Guatemala City, Guatemala and Supplementary Table 1), which is enriched in protein, vitamins and other compounds provided to children in Guatemala kindergartens and approved by the Institute of Nutrition of Central America and Panama (INCAP). To perform the tests, the protocol described by Haffner et al. (2017) was adapted to this study (see details in Figure 1). The food matrix was dissolved in water and heated in a microwave oven following the directions of the product. Then, 10^8^ cfu mL^−1^ of each LAB were resuspended independently in saline solution (1 mL), and each one used to inoculate 30 mL of the matrix. These bacterial suspensions were then subjected to the following consecutive exposures to stresses at 37°C with shaking (100 rpm):

- gastric phase, the pH of samples inoculated were gradually decreased from 6.5 (initial pH of the food matrix) to 2.0 with 2.5 M HCl, with intervals of 0.5–1.0 units and with 15 min incubation at each pH. Aliquots of 200 μL were withdrawn prior to each pH decrease;
- small intestine phase, first the pH was increased with 2.5 M NaOH from 2.0 to 4.0. then, 10 mL of pancreatic and bile juice solution [0.6% w/v of bile salts (Sigma-Aldrich), 1.25% w/v of NaHCO_3_ and 0.09% w/v of pancreatin (Sigma-Aldrich)] were added to the gastric phase, generating a pH increase to 7.0, and the mixture was incubated for 3 h. Aliquots of 200 μL were withdrawn after each hour.

Finally, 100 μL of the aliquots subjected to the treatments in adequate dilutions were streaked on MRS plates, incubated at 37°C for 48 h, and the number of cfu was calculated for each condition evaluated by plate counts. The results were expressed as percentage of surviving cells (initial bacterial concentration = 100%).

### *In vivo* Animal Trials

Conventional adult BALB/c mice (male, 4 weeks old, weighing 12 ± 2 g) were obtained from the animal facility of the Centro de Referencia para Lactobacilos (CERELA-CONICET, San Miguel de Tucumán, Argentina) and were maintained in ventilated cages under controlled conditions (18–20°C and a 12 h light/dark cycle). The animal protocol was approved by the Animal Protection Committee of CERELA (protocol no. CRL-BIOT-LT-20142/A), and all experiments complied with the current laws of Argentina for the use of experimental animals.

**Figure 2:**
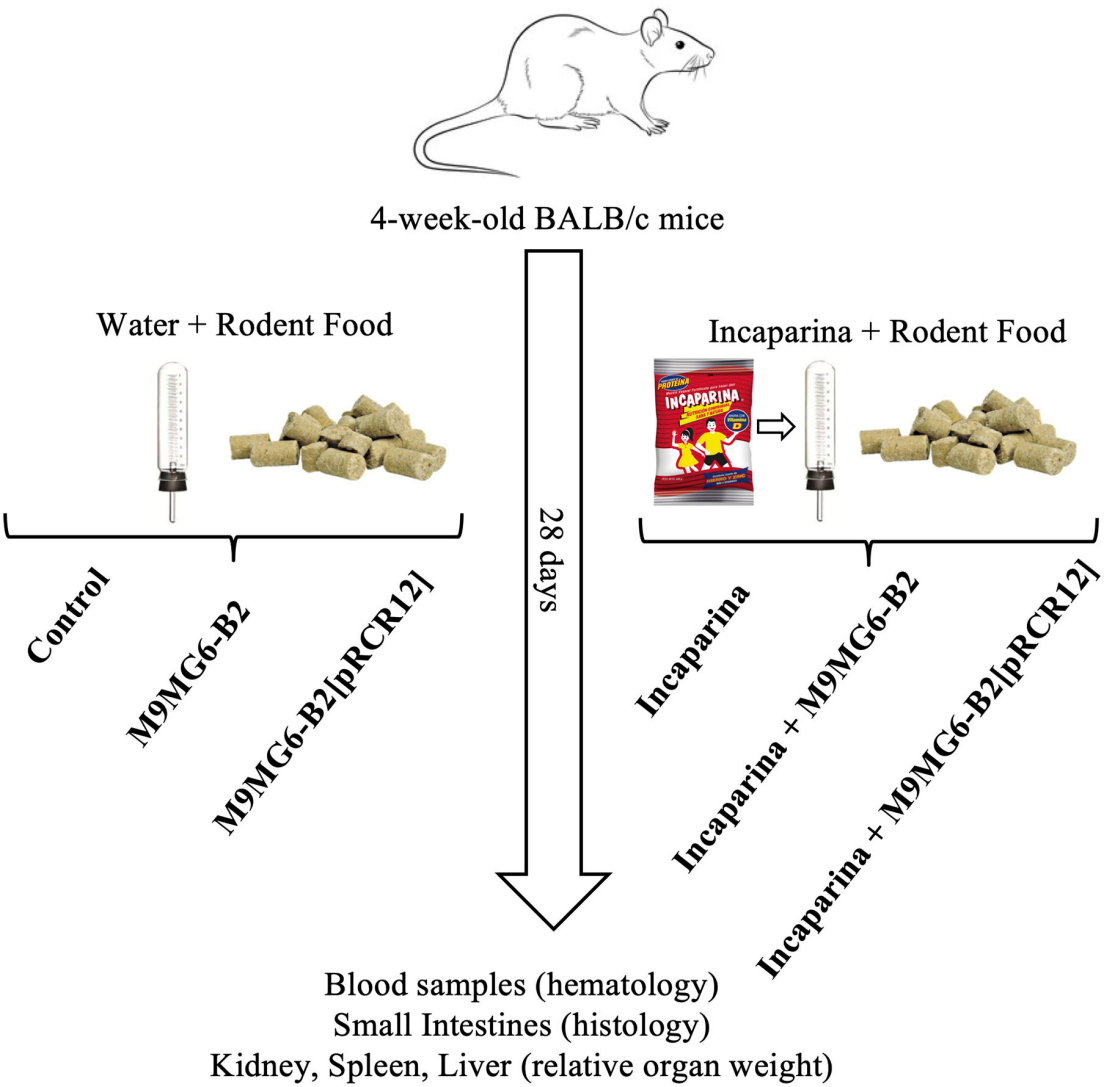
Experimental animal protocol. Balb/c mice were divided into groups that received water and rodent food without bacterial supplementation (control group), that were supplemented with *L. plantarum* M9MG6-B2 (M9MG6-B2 group) or *L. plantarum* M9MG6-B2[pRCR12] [M9MG6-B2[pRCR12] group] or that received Incaparina and rodent food without bacterial supplementation (Incaparina group), supplemented with *L. plantarum* M9MG6-B2 (Incaparina + M9MG6-B2 group) or *L. plantarum* M9MG6-B2[pRCR12] [Incaparina + M9MG6-B2[pRCR12] group] during 28 days. Then, the animals were sacrificed and blood samples taken for hematological studies, small intestines used for histological evaluations and organs (kidney, spleen, and liver) used to determine relative organ weights.

Animals were divided into 6 experimental groups consisting of at least 6 mice each (see protocol in Figure 2). The Control group did not received any special treatment, and were fed with balanced conventional rodent food and diluted milk (1:30 low fat milk/water, v:v) *ad libitum*. The bacterial supplemented groups received M9MG6-B2 or M9MG6-B2[pRCR12] strains in diluted milk at a concentration of 1 × 10^9^ cfu mL^−1^. To this end, strains were previously grown in MRS medium for 16 h at 37°C, sedimented (5,000 × *g*, 10 min, 4°C) washed with saline solution, resuspended in the original volume with low fat milk and diluted in water. The animals that were fed with Incaparina received the product as a replacement of their drinking water. In the groups that received both Incaparina and bacterial supplementation [Incaparina + M9MG6-B2 and Incaparina + M9MG6-B2[pRCR12] groups], the bacterial strains were prepared as stated above and resuspended in Incaparina at a concentration of 1 × 10^9^ cfu mL^−1^, and this mixture was given as a replacement of water. These supplements/water replacements were provided to the animals for 28 days (each drinking bottle was replaced twice daily); food and water/Incaparina consumption was measured daily. Animal live weights and behavior were determined on a bi-daily basis. Feces were collected every 5 days from the animals that consumed M9MG6-B2[pRCR12] in the presence or absence of Incaparina. These samples were homogenized and diluted in sterile saline solution and plated on MRS-agar plates containing Cm. Pink colonies, consisting of the strain that expressed mCherry were counted. At the end of the experimental period (28 days), animals were euthanized with an intraperitoneal injection of ketamine (Holliday, Scott S. A.) and xylazine (Rompun, Bayer S. A.) to obtain a final concentration of 100 and 5 μg/kg live body weight, respectively. Blood samples were taken by cardiac puncture and transferred into a tube containing EDTA as anticoagulant (1.5 mg mL^−1^ of blood) for hematological studies. The intestines of the animals were extracted, and the content removed with saline solution, cut into three parts and transferred separately to tubes containing 10% formaldehyde for histological analysis. Spleen, kidneys, and liver were excised, washed, air dried and weighed.

### Histological Analysis of the Small Intestines

Fixed intestines were embedded in paraffin, sectioned (4 μm) and colored with hematoxylin and eosin. Measurements of small intestine villi length and crypts depth were performed using an optical microscope (Carl Zeiss-Axio Scope-A1, Germany), with a 100 × magnification lens. The images were analyzed using AxioVision Release 4.8 software, calculating the villus length/crypt depth ratio (5 measurements per animal).

### Statistical Analysis

All experiments were performed in triplicate. The results are expressed as the mean and the corresponding standard deviation. For the adhesion, auto-aggregation, and biofilm formation assays, in order to establish differences between strains, the data were subjected to one-way analysis of variance (ANOVA). Mean pairwise comparisons were computed with a Tukey's test with a *p* ≤ 0.05. For analysis of the LAB survival cells (%) after the gastric and intestinal stresses, in the cases where strain and condition interaction was significant, *t*-tests were performed to determine if strains were significantly different at each condition level with a *p* ≤ 0.05. All analyses were performed with the R software version 4.0.0 (R Core Team., 2020).

For the *in vivo* animal trials, data were assessed using the ANOVA general linear model, with the level of significance at *P* ≤ 0.05. The results are presented as mean ± standard deviation (SD). Significant differences between groups were determined by Tukey's *post-hoc* test. Statistical analyses were performed using MINITAB 15 software (Minitab, State College, PA, USA).

## Results and Discussion

### Analysis of the Effect of mCherry Labeling on the Growth of *L. plantarum* M5MA1-B2 and M9MG6-B2 and on Riboflavin Production

Plasmid pRCR12 encodes the mCherry protein, and previous construction and analysis of *L. plantarum* M5MA1-B2[pRCR12] revealed that the fluorescence conferred by the plasmid allows discrimination of the bacterium in a digestive tract microbiota context and also quantitative assessment of bacterial adhesion to Caco-2 cells (Mohedano et al., 2019). Therefore, in this current work we aimed to standardize new fluorescence-based quantification techniques to evaluate probiotic characteristics of *L. plantarum* strains not previously analyzed. To this end, after the construction of *L. plantarum* M9MG6-B2[pRCR12] by plasmid transfer, we compared the performance of the riboflavin-overproducing M5MA1-B2 and M9MG6-B2 strains and their isogenic labeled strains. As expected, M5MA1-B2 and M9MG6-B2 grown in MRS-agar medium developed white colonies, while M5MA1-B2[pRCR12] and M9MG6-B2[pRCR12] showed the characteristic pink color that indicates the functional expression of the mCherry protein (Supplementary Figure 3A). Likewise, red fluorescence was detected by fluorescence microscopy in M5MA1-B2[pRCR12] and M9MG6-B2[pRCR12] cultures grown in MRS broth, but not observed in cultures of the isogenic unlabelled parental strains (Supplementary Figure 3B).

Also, in cultures grown in MRS broth the bacterial growth and the mCherry fluorescence were monitored simultaneously during 14 h (results not shown) and from these data the growth rate (μ) and doubling time (dt) of the four LAB during the exponential phase of growth were calculated, as were the levels of the fluorescence at the end of the assays (Table 2). For M5MA1-B2 and M9MG6-B2 strains a μ value of 0.56 ± 0.02 h^−1^ was observed with a corresponding dt of 1.24 ± 0.04 h. A lower μ was observed for the labeled strains, 0.48 ± 0.01 h^−1^ for M5MA1-B2[pRCR12] vs. 0.46 ± 0.01 h^−1^ for M9MG6-B2[pRCR12], with a concomitant increment in the doubling time, 1.45 ± 0.04 h vs. 1.52 ± 0.04 h, respectively. Expression of the mCherry in cultures of the two strains carrying pRCR12 plasmid, was confirmed since 100 au of fluorescence were recorded, while in those of the isogenic M5MA1-B2 and M9MG6-B2 only about 1 au of background fluorescence was detected.

**Table 2:** Riboflavin production, mCherry expression and kinetic growth parameters of *L. plantarum* riboflavin-overproducing strains.

**Strain**	**MRS medium**			**CDM medium**						
	**μ (h^**−1**^)**	**dt (h)**	**mCherry fluorescence (au)**	**μ (h^**−1**^)**	**dt (h)**	**Fluorescence (au)**	**Riboflavin concentration (mg L^**−1**^)**	**OD_**480 nm**_**	**Specific concentration of riboflavin^a^**	**Mutation in the RFN region**
M5MA1-B2	0.56 ± 0.02	1.24 ± 0.03	1.01 ± 0.07	0.52 ± 0.01	1.34 ± 0.01	25.34 ± 2.84	3.19 ± 0.42	4.0	0.80	G19A
M9M6G-B2	0.56 ± 0.01	1.24 ± 0.03	1.08 ± 0.10	0.55 ± 0.01	1.25 ± 0.03	21.23 ± 1.74	2.78 ± 0.22	3.7	0.75	G19C
M5MA1-B2 [pRCR12]	0.48 ± 0.01	1.45 ± 0.04	94.63 ± 3.8	0.34 ± 0.01	2.06 ± 0.07	25.54 ± 6.68	2.59 ± 0.27	3.2	0.81	G19A
M9MG6-B2 [pRCR12]	0.46 ± 0.01	1.52 ± 0.04	101.80 ± 3.17	0.40 ± 0.01	1.72 ± 0.05	24.37 ± 6.74	3.12 ± 0.87	4.6	0.68	G19C

*All values are expressed as means ± standard deviation of three independent experiments. Values referred to riboflavin, mCherry and D_480 nm_ correspond to cultures in the stationary phase after 14 h of growth in the indicated medium. The growth rate (μ) and doubling time of these cultures during the exponential phase is also depicted*.

a *Specific concentration of riboflavin = Riboflavin concentration/OD_480 nm_*.

We have previously validated quantification of riboflavin levels by determination of vitamin B2 fluorescence during the *L. plantarum* growth in the non-fluorescent CDM-Rib medium deprived of the vitamin (Mohedano et al., 2019). Thus, this method was used to determine production of riboflavin by monitoring simultaneously the growth and the vitamin fluorescence in cultures grown in CDM-Rib medium. The substitution of MRS by CDM-Rib as growth medium had little effect on the M5MA1-B2 and M9MG6-B2 μ and dt parameters (Table 2). However, a clear negative effect was observed in the case of the pRCR12 plasmid-carrier strains, with a decrease in μ values from 0.48 to 0.34 h^−1^ for M5MA1-B2[pRCR12] and from 0.46 to 0.40 h^−1^ for M9MG6-B2[pRCR12], accompanied by an increase in the doubling time from 1.45 to 2.06 h and from 1.52 to 1.72 h, respectively. For all four strains, vitamin synthesis was directly associated with the exponential phase of growth, reaching its maximum during this phase and being practically constant throughout the stationary phase (results not shown). After the 14 h incubation period, 25 au of fluorescence were recorded for the M5MA1-B2 and M5MA1-B2[pRCR12] strains, whereas for the M9MG6-B2 and M9MG6-B2[pRCR12] the results were 20 and 24 au, respectively (Table 2). When fluorescences were expressed as riboflavin concentration, they ranged from 2.6 mg L^−1^ for M5MA1-B2[pRCR12] to 3.19 mg L^−1^ for M5MA1-B2 (Table 2), high values which were similar to those obtained in previous results (Mohedano et al., 2019). Also, when the specific concentration of vitamin B2 was estimated (considering the optical density of the cultures as reference of the cellular biomass), the values for M5MA1 and M5MA1-B2[pRCR12] were almost identical 0.80 and 0.81 and for M9MG6-B2 and M9MG6-B2[pRCR12], only slightly lower 0.75 and 0.68, respectively (Table 2).

Thus, the results obtained showed that the labeling with the plasmid pRCR12 did not significantly affected the production of riboflavin. In addition, the burden of carrying the plasmid only moderately affected the growth of the strains, corroborating our previous observations with *L. plantarum* 90, B2 and M5MA1 strains (Russo et al., 2015; Mohedano et al., 2019) or *L. sakei* MN1 (Nácher-Vázquez et al., 2017) carrying the pRCR12 plasmid; thus, supporting the suitability of this vector in LAB for future studies with fluorescent labeling.

### Interactions of Labeled and Unlabeled *L. plantarum* M5MA1-B2 and M9MG6-B2 Strains With Human Enterocytes

**Figure 3:**
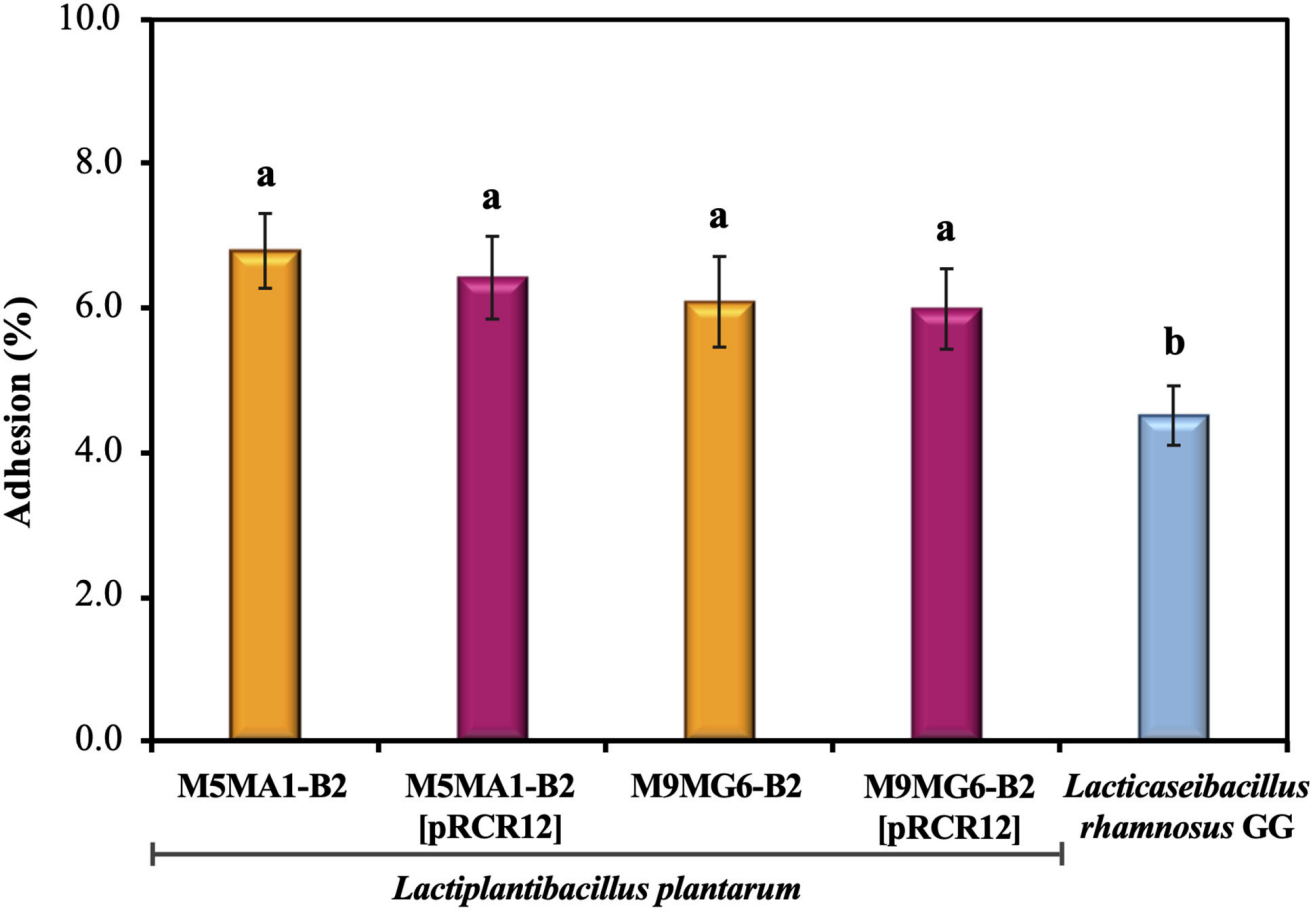
Adhesion of the LAB strains to the biotic Caco-2 human cell line. The assays were performed during 2 h. Adhesion levels are expressed as the percentage of cfu bound. Hundred percentage corresponds to the number of bacteria added to the Caco-2 cells. The ANOVA statistical analysis of the results is depicted. A *p* ≤ 0.05 was considered significant. The Tukey's test was employed (α = 0.05) to test the statistically significant differences between samples. Means with the same letter were not significantly different.

After ingestion of probiotic bacteria, their survival and persistence in the gut depend on host–bacteria interactions. Successful adhesion of probiotic bacteria to the gut epithelium has been reported to help establish colonization in the gut (Gueimonde and Salminen, 2006). As a first approach to test bacterial binding to enterocytes, human colon cell lines e.g., Caco-2 are used, due to the difficulty of examining the process *in vivo* (Kim et al., 2015). This cell line differentiates spontaneous to enterocyte-like cells after reaching confluence and expresses characteristics of mature enterocytes, including polarization, brush border and apical intestinal hydrolases (Pinto et al., 1983). Thus, the adhesion to Caco-2 cells of the four *L. plantarum* strains in comparison with that of the probiotic *L. rhamnosus* GG was investigated (Figure 3). The assays were performed with 1.0 × 10^5^ bacteria exposed to 1.0 × 10^4^ Caco-2 cells, with the aim to not saturate the binding capacity of the enterocytes in order to unmask differences between the strains. The levels of adhesion were very similar for the four *L. plantarum* strains analyzed, ranging from 6.0 to 6.81%. These results showed that both riboflavin-overproducing strains showed the same behavior and that the presence of the pRCR12 did not affect negatively the capability of any of the four bacteria to interact with the biotic surface. Moreover, *L. rhamanosus* GG displayed a 4.51% ± 0.41 adhesion level, slightly lower than that of the four *L. plantarum* under study. These adhesion levels highlight the probiotic potential of the M5MA1-B2 and M9MG6-B2 strains. In addition, the detected adhesion levels of M5MA1-B2 and M5MA1-B2[pRCR12] (6.81% ± 0.52 and 6.42% ± 0.57) were ~16-fold higher than that obtained previously (0.48% ± 0.07 and 0.34% ± 0.07 for the unlabelled and labeled strains, respectively) (Mohedano et al., 2019). This discrepancy was expected, since the levels of binding were expressed as percentage of the added bacteria, and in our previous work the adhesion evaluation was performed using an over-saturation proportion of 5 × 10^9^ bacteria to 1 × 10^5^ Caco-2 cells. Supporting the results obtained here, studies performed with other *L. plantarum* strains isolated from both fermented foods and beverages showed adherence values similar to those obtained in the present study. Under similar experimental conditions to the ones performed here, an adhesion value to Caco-2 cells of 12.2%, 10.2% and 8% were reported for *L. plantarum* L15 (Alizadeh Behbahani et al., 2019), Lp91 (Duary et al., 2011) and 423 (Botes et al., 2008) strains isolated from cereal-dairy product, human fecal sample and sorghum beer, respectively.

### Biofilms Formed by Labeled and Unlabeled *L. plantarum* M5MA1-B2 and M9MG6-B2 Strains on an Abiotic Surface

In addition to having the ability to bind to intestinal cells, probiotics should be able to form biofilms, a property that might provide the bacteria with protection against adverse environmental conditions, and consequently to promote their survival and persistence in the intestinal environment. Thus, to determine the capability of M5MA1-B2 and M9MG6-B2 to form biofilm, the crystal violet staining method was used (see Materials and Methods section Crystal Violet Staining Method) with polystyrene multi-well plates as the abiotic support (Table 3). The measurement of the OD_570 nm_ of the stained biofilms of M5MA1-B2 and M9MG6-B2 gave values of 3.84 ± 0.31 and 3.52 ± 0.17, the former being slightly but significantly higher than the latter (*p* ≤ 0.05). In addition, these values were 39- and 44-fold higher than the value of the control sample (0.09 ± 0.17). Therefore, these results revealed that, according to the classification established by Hashem et al. (2017), the two *L. plantarum* strains, are very superior to the category of “strongly adherent or biofilm-forming” that only requires OD_570 nm_ of the samples 4-fold higher than that of the control (see details of categories in Material and Methods section Crystal Violet Staining Method). Detection of biofilm formation by the crystal violet staining method has been widely used for various bacterial species because the dye binds non-specifically to cells and matrix components, making it a high throughput method to quantify the total biomass that forms the biofilm (Merritt et al., 2005). However, parameters such as medium composition, temperature, and time of incubation may affect the biofilm quantification and interfere with the validity of this method. Thus, a study carried out with *L. plantarum* WCFS1 and other strains showed that the OD_570 nm_ of the biofilm was higher at 30 or 37°C vs. 20 or 25°C, but caused a reduction in the number of living cells (Fernández Ramírez et al., 2015). Similarly, in the same study a prolonged incubation time (24–48 h) caused an increase in the number of cells that make up the biofilm, but after 72 h, although higher values of OD_570 nm_ were detected, the viable cells decreased. These results were interpreted by the authors as indicating that the crystal violet may also bind to dead cells, extracellular DNA (eDNA), proteins, exopolysaccharides, and other intracellular components, which could interfere in biofilm quantification, thereby overestimating the number of viable cells that constitute the biofilm matrix. Therefore, in this work, we have standardized a fluorometric method to quantify biofilm formation on polystyrene multi-well plates as abiotic support by means of the intracellular fluorescence emitted by the mCherry protein (see details in Materials and Methods section Fluorometric Method by mCherry Fluorescence Measurement). The cultures used to generate the biofilms (1.0 × 10^6^ cfu) showed an initial fluorescence of 1–2 au, while the biofilms of M5MA1-B2[pRCR12] and M9MG6-B2[pRCR12] formed after incubation for 48 h exhibited similar levels of fluorescence (102.60 ± 6.20 au vs. 99.94 ± 7.8) (Table 3). Moreover, these levels corresponded to ~1.8 × 10^8^ cfu and 1.4 × 10^8^ cfu mL, respectively, values estimated using the calibration curve of each strain presented in Supplementary Figure 2. Thus, the increment of more than two log_10_ units in the cfu mL^−1^ demonstrated an efficient biofilm formation capability of the M5MA1-B2[pRCR12] and M9MG6-B2[pRCR12] strains, correlating with the qualitative estimation of “strongly adherent” obtained by the violet crystal staining method for their parental unlabeled strains, leading us to consider the method implemented in this work as valid for future quantifications.

**Table 3:** *In vitro* adhesive properties of riboflavin-overproducing strains on an abiotic surface.

**Strain**	**Biofilm determination**		**Auto-aggregation**
	**Methods**		
	**Crystal violet**	**mCherry**	**mCherry**
	**staining**	**fluorescence**	**fluorescence**
	**(OD_**570 nm**_)**	**(au)**	**method (%)**
Ctr^1^	0.09 ± 0.17^c^	1.25 ± 0.35^b^	100^a^
M5MA1-B2	3.84 ± 0.31^a^	ND	ND
M9MG6-B2	3.52 ± 0.17^b^	ND	ND
M5MA1-B2[pRCR12]	ND	102.6 ± 6.3^a^	29.13 ± 4.0^b^
M9MG6-B2[pRCR12]	ND	99.94 ± 7.8^a^	34.68 ± 3.9^a^

*Values are expressed as means ± standard deviation for n = 3 independent experimental assays*.

a, b, c Means with different letter differed significantly (p ≤ 0.05).

ND, not determined.

1 *Ctr corresponds to either: (i) the values of OD_570 nm_ or mCherry fluorescence obtained for the biofilms formed with only medium instead of bacteria or (ii) the initial fluorescence (100%) of the 2 × 10^7^ cfu used in the auto-aggregations assays*.

### Auto-aggregation Capability of Labeled *L. plantarum* M5MA1-B2 and M9MG6-B2 Strains

The ability of probiotic bacteria to form cellular aggregates via self-aggregation (auto-aggregation) or between genetically distinct cells (co-aggregation) is a desirable characteristic. Auto-aggregates can adhere to surface mucosa increasing probiotic persistence in the intestine where they can potentially inhibit the adherence of pathogenic bacteria, and prevent their colonization (Monteagudo-Mera et al., 2019). According to Abdulla et al. (2014), this property also determines the ability of a probiotic to adhere to other surfaces such as the oral cavity, and the gastrointestinal and urogenital tracts. Since the usage of mCherry fluorescence measurement seemed to be a useful tool to assess the probiotic properties of *L. plantarum* (Mohedano et al., 2019) and above, we decided to expand the usage of the protein in this work to a method that allows the determination of the degree of bacterial auto-aggregation by measuring the fluorescence emitted by bacterial cultures (see details in Materials and Methods section 1.8). The method was used to establish that both labeled riboflavin-overproducing strains possess auto-aggregative profiles (Table 3). After 15 h of incubation at 37°C, an auto-aggregation value of 34.68 ± 3.9% for M9MG6-B2[pRCR12] strain was obtained, a value slightly, but statistically significantly higher than the 29.13 ± 4.0% detected for the M5MA1-B2[pRCR12] strain. The methodology used here to assess auto-aggregation has not been previously described, thus impairing our ability to make comparisons with the observations of other researchers. However, by means of a spectrophotometric technique, the aggregative profile of various LAB strains has been characterized. In particular, high percentages of auto-aggregation have been reported for *Lactiplantibacillus* strains: 28.27 ± 9.7% for *L. plantarum* 46a (Raveschot et al., 2020); around a 40 % for *L. plantarum* L15 (Alizadeh Behbahani et al., 2019), and within a range of 29.47 ± 2.63% to 33.57 ± 2.21% in other *L. plantarum* strains. Therefore, our results seem to be those expected for *L. plantarum* strains and indicate a good auto-aggregation capability of M5MA1-B2 and M9MG6-B2, which could have a certain relationship with their good adhesion to the biotic Caco-2 cells surface and the high capacity to form biofilms on abiotic surfaces, characteristics that would give them certain advantages to colonize the intestine. Similar observations have been described by several authors who have established that the ability of bacteria to aggregate seems to have an important effect on biofilm formation, and therefore this ability is related to the cell adherence properties, and the ability to survive and persist in the GIT (Vlková et al., 2008; Ferreira et al., 2011). In correlation with the above stated, some lactobacilli have been described to have an aggregation phenotype. However, the mechanisms of cellular aggregation have not been fully elucidated and may be species specific and environment dependent (García-Cayuela et al., 2014). Certain authors have proposed a correlation between auto-aggregation and hydrophobicity, so that high auto-aggregation values showed also high hydrophobicity (Nikolic et al., 2010). However, other authors did not observe such a correlation, which indicates that interaction of probiotics with intestinal cells involves more complex mechanisms (García-Cayuela et al., 2014). Similarly, Ramiah et al. (2008) reported that factors other than surface-bound proteins play an important role in adhesion. The glycoproteins, teichoic and lipoteichoic acids on the cell wall surface of bacteria contribute to the adhesion, auto-aggregation abilities, and hydrophobicity of the strains (Darilmaz et al., 2012; Haddaji et al., 2015). Several proteins involved in bacterial aggregation or biofilm formation have been described such as Esp protein (~206 kDa) in *Enterococcus faecalis* that promotes adhesion and biofilm formation (Toledo-Arana et al., 2001) or Bap protein (~250 kDa) in *Staphylococcus aureus* involved in biofilm formation (Cucarella et al., 2001). Hence, it is possible that the M5MA1-B2 and M9MG6-B2 riboflavin-overproducing strains may interact with epithelial cells of the host with a temporary colonization on it. In this way they might exert some beneficial effects, due to the considerable high adhesive profile probed in this study. According to Monteagudo-Mera et al. (2019) a temporary colonization facilitates the local action of metabolites produced by probiotics, such as short-chain fatty acids, as well as immunomodulatory effects.

### Resistance to GIT Stresses of Labeled and Unlabeled *L. plantarum* M5MA1-B2 and M9MG6-B2 Strains Included in a Food Matrix

**Figure 4:**
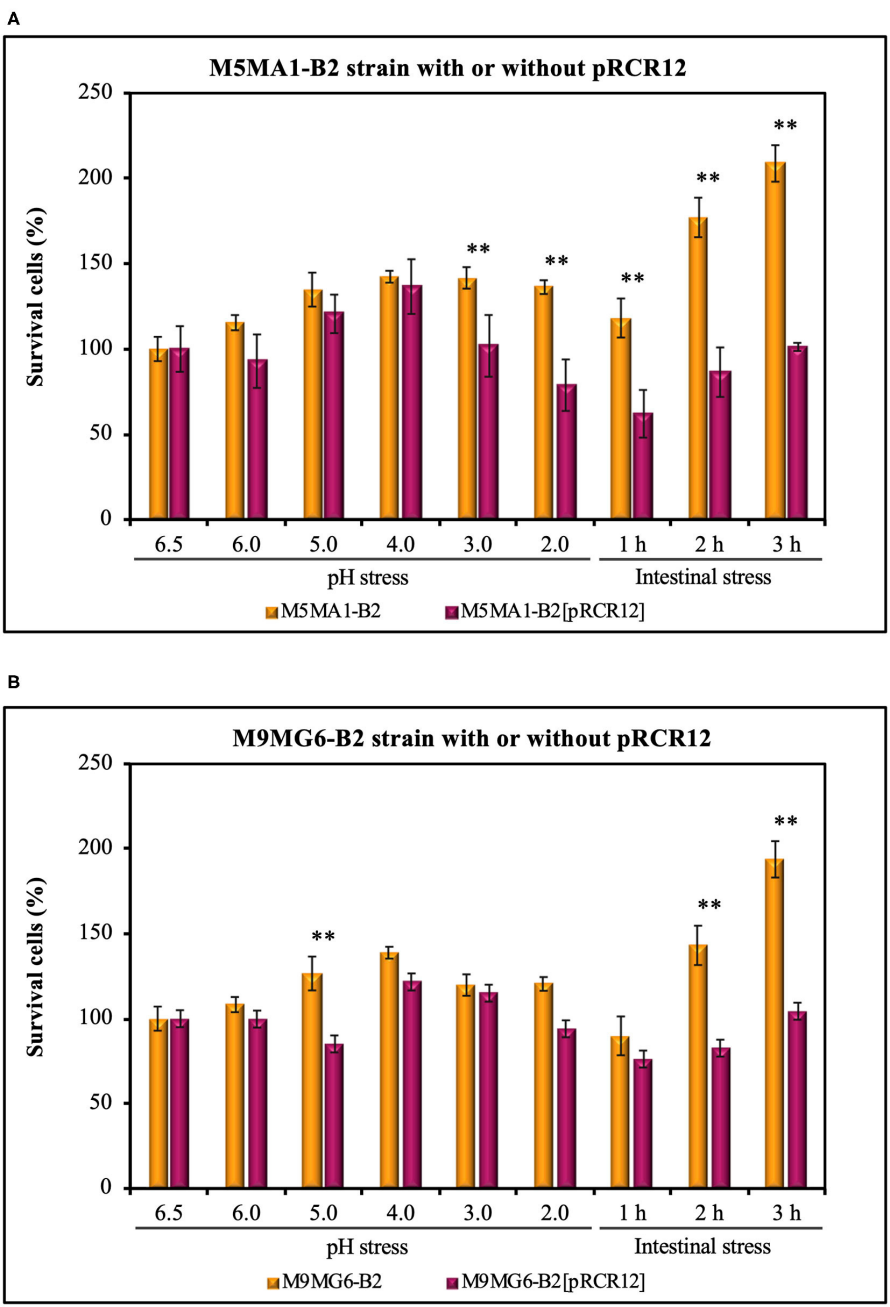
Analysis of resistance to gut stresses of the LAB strains: M5MA1-B2 and M5MA1-B2[pRCR12] **(A)** as well as M9MG6-B2 and M9MG6-B2 **(B)** in a food matrix. The bacteria were subjected to acidic (pH) or intestinal stresses as described in Figure 1. Cell survival was determined by plate counting and expressed as percentage. The values are the mean of three independent experiments and are expressed as a total percentage of surviving cells compared to the initial bacterial population. A statistical *t*-test analysis of the results is depicted. A *p* ≤ 0.05 was considered significant. Strains with **are significantly different at each condition tested.

The acidic environments found in some foods, and in the stomach provide a survival challenge for probiotic microorganisms. Predominantly, the delivery vehicles for probiotic cultures have been yogurts and fermented milks, which themselves present an acid environment. Then, the probiotics must survive the highly acidic gastric juice in order to reach the small intestine in a viable state (Henriksson et al., 1999). Hence, gastric acid stress is a key test for their survival (Ranadheera et al., 2014). Additionally, a probiotic strain must pass through stress challenges in the GIT including tolerance to the bile salts in the upper parts of the small intestine (Begley et al., 2005). Thus, we have evaluated the capacity of the *L. plantarum* strains to resist simulated stress conditions following the protocol depicted in Figure 1. Previous work revealed that the parental strains isolated from chicha have good technological properties in cereal-based food matrices (Yépez et al., 2019). Thus, the LAB were suspended in a non-acidic food matrix, Incaparina, which is a cereal-based beverage administrated in children's day care services in Guatemala, and cell viability was determined after each stress (Figure 4). The initial bacterial concentration in the *in vitro* assay was considered as 100% and corresponded to 1.0 × 10^8^ cfu mL^−1^. The four *L. plantarum* strains were subjected to a decreasing pH gradient from 6.5 to 2.0 by sequential incubations at different pHs. These conditions were chosen, because humans secrete, on average, 2.5 L of gastric juice per day, resulting in a fasting gastric pH value of 1.5, which increases to between pH 3.0 and 5.0 during feeding (Hill, 2002). Also, many studies have reported that a value of pH <2.0 could destroy bacteria; however, this pH rarely occurs in the stomach (Alizadeh Behbahani et al., 2019). The results obtained here show that the two parental strains M5MA1-B2 (Figure 4A) and M9MG6-B2 (Figure 4B) did not lose any viability during the exposure to the gastric stress. In addition, the number of cfu mL^−1^ increased after exposure to pH 4 to a maximum value of 142 or 139%, with 136 or 121% still remaining at pH 2 for strains M5MA1-B2 or M9MG6-B2, respectively. In the case of M5MA1-B2[pRCR12] (Figure 4A) and M9MG6-B2[pRCR12] (Figure 4B) an initial increase of viability was also observed for both strains with maximum values at pH 4 of 137 and 122%, respectively. In addition, the viability of M5MA1-B2[pRCR12] significantly decreased to 102% at pH 3 and to 79% at pH 2, whereas this loss of survival was less for M9MG6-B2[pRCR12], with values of 115 and 94% at pH 3.0 and 2.0, respectively. Thus, the results show a high capacity of the four strains to survive in the stomach environment. This acid resistance could be intrinsic, as has been previously detected in other lactobacilli (de Angelis and Gobbetti, 2004; Wall et al., 2007; Broadbent et al., 2010). In Gram-positive microorganisms, including LAB, F_0_F1-ATPase is one of the main mechanisms studied for protection against acidic conditions. F_0_F_1_-ATPase is induced at low pH and can increase the intracellular pH under acidic environmental conditions (Cotter and Hill, 2003). In addition, other mechanisms such as decarboxylation and deamination, cell membrane modification and macromolecule protection and repair have been proposed as alternative processes to mediate LAB acidic response (Guan and Liu, 2020).

The results obtained in this work also indicated an important role of the food matrix in the bacterial performance. The viability of the bacteria did not decrease, since quantification of bacteria by plate count revealed an increase of the cfu mL^−1^, even during incubation at pH 4.0, indicating that the components of the food matrix were metabolized by the LAB and supported their growth. Furthermore, under any of the acid stress conditions, the viability of any of the LAB tested was higher than 21%, which could be due to a protective effect of the evaluated food matrices. Since the matrix has high viscosity, it might promote cellular aggregation that could be the first step of biofilm formation. The high auto-aggregation and biofilm formation ability of the strains detected here (Table 3) support this hypothesis. Likewise, it has been proposed for other LAB that formation of biofilms protects microbial cells against acidic shock. Thus, cell density, related to the formation of biofilms, is another factor affecting the acid resistance of microorganisms (Liu et al., 2015) and could be involved in the acid resistance detected for the M5MA1-B2 and M9MG6-B2 strains.

After feeding, bile is released into the duodenum and acts in the emulsification and solubilization of lipids in the small intestine. It can also destroy the phospholipids and proteins of bacterial cell membranes. Thus, a highly desirable quality of a probiotic strain is its ability to survive in the presence of bile salts in the upper parts of the small intestine (Begley et al., 2005). Thus, after, exposure to stomach stress, the *L. plantarum* strains were subjected to intestinal stress (Figure 4). The LAB were treated at pH 7.0 with bile salt and pancreatic juice containing protease, lipase and amylase. The exposure to this environment generated some deleterious effects on the viability of the four *L. plantarum* strains during the first hour of treatment, with recovery of cfu during the subsequent 2 h of incubation (Figure 4). A clear and significant 2-fold difference in bacterial survival was observed between, M5MA1-B2 and M5MA1-B2[pRCR12] (Figure 4). After 1 h of intestinal stress challenge, viability of the former remained at 119% of the initial values, whereas the latter showed only 62% survival (Figure 4A). This difference was also observed after 2 and 3 h of treatment, when M5MA1-B2 showed a viability of 177 and 209% vs. 86% and 101% for M5MA1-B2[pRCR12] (Figure 4A). This lower tolerance of M5MA1-B2[pRCR12] was also observed after challenge at pH 3.0 and 2.0 with 1.4- and 1.7-fold greater decrease of viability than M5MA1-B2 (Figure 4A). The presence of pRCR12 in M9MG6-B2 under intestinal stress for 1 h had no significant effect (Figure 4B), as was detected after acidic stress at pH 3.0 and pH 2.0, where, under the latter condition M9MG6-B2[pRCR12] was only slightly (1.2-fold) more sensitive than M9MG6-B2. However, the recovery after 2 and 3 h of intestinal treatment was higher for the unlabeled parental (143 and 194%) than the labeled (83 and 109%) strain (Figure 4B).

### *In vivo* Animal Trials

The *in vitro* results presented above support the good probiotic potential of the riboflavin-overproducing M5MA1-B2 and M9MG6-B2 strains, and to Incaparina as a suitable food matrix to protect these LAB against the GIT stresses, as well as to promote their delivery to the intestine and therefore it supports their proliferation. However, there was not a clear-cut difference between the performances of these two strains. Therefore, M9MG6-B2 and M9MG6-B2[pRCR12] in the presence or absence of Incaparina were further tested in an *in vivo* murine model using BALB/c mice, evaluating growth parameters of the animals as well as possible changes in particular organs following the scheme depicted in Figure 2. All the animals started with similar initial weights. After the experimental period (28 days), no differences were observed in the animal weight increase, or in the relative weight of the kidneys, spleens, and liver between all the experimental groups (Table 4). These results demonstrate that bacterial supplementation by itself or included in the Incaparina matrix did not significantly affect animal growth. Similarly, hematological analysis did not show significant differences between the groups. White and red blood cells' counts, relative differential leukocytes, hemoglobin were maintained between the reference values for these animals (data not shown).

**Table 4:** Influence of *L. plantarum* M9MG6-B2 or M9MG6-B2[pRCR12] strains supplementation with or without Incaparina on BALB/c mice and their organs weights.

**Group**	**Initial weight^a^**	**Weight increase^b^**	**Kidney/animal**	**Spleen/animal**	**Liver/animal**
	**(g)**	**(g)**	**weight^c^ (%)**	**weight^c^ (%)**	**weight^c^ (%)**
Control	13 ± 1	17 ± 2	1.5 ± 0.1	0.3 ± 0.1	5.0 ± 0.4
Incaparina	13 ± 2	16 ± 3	1.5 ± 0.2	0.3 ± 0.1	5.4 ± 0.4
M9MG6-B2	13 ± 2	17 ± 2	1.5 ± 0.1	0.3 ± 0.1	5.4 ± 0.3
M9MG6-B2[pRCR12]	12 ± 1	18 ± 2	1.5 ± 0.2	0.3 ± 0.1	5.3 ± 0.5
Incaparina + M9MG6-B2	13 ± 1	16 ± 2	1.5 ± 0.1	0.3 ± 0.1	5.0 ± 0.3
Incaparina + M9MG6-B2 [pRCR12]	12 ± 2	17 ± 3	1.4 ± 0.1	0.4 ± 0.1	5.1 ± 0.5

a *Initial weights of animals measured previous to starting the experiment*.

b *Weight increase was calculated by determining the difference between the animals' weights before and after the experiment (day 0 vs. day 28)*.

c *Kidney, spleen and liver weights were divided by animal live weight at day 28*.

*Data are expressed as means ± standard deviation for n = 6 animals in each group. A total N = 36 animals were used in the experiment)*.

**Figure 5:**
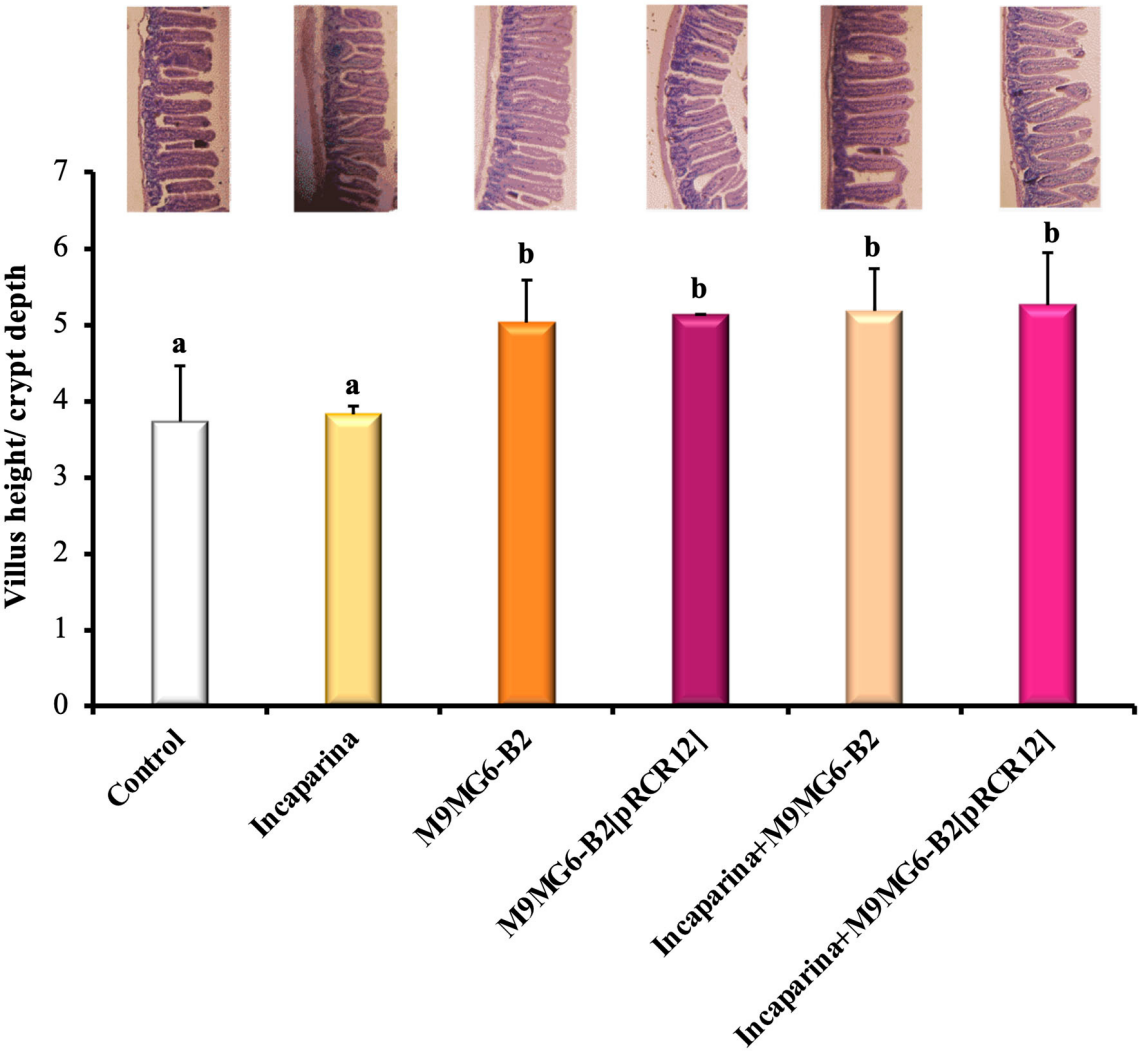
Histological evaluation of the influence of *L. plantarum* M9MG6-B2 supplementation on the morphology of BALB/c mice small intestines. The organs of mice from the Control group, and those who received bacterial supplementation with either *L. plantarum* M9MG6-B2 or M9MG6-B2[pRCR12] strains with or without Incaparina were evaluated The ratio of the villus height/crypt depth is shown. ^a,b^Columns with different letters are significantly different between each other with a *p* ≤ 0.05. On top of each column is a representative photograph of each experimental group.

The maintenance of intestinal mucosa integrity requires coordination of the cell proliferation and differentiation processes (Parker et al., 2017). The analysis of intestine revealed that the administration of Incaparina did not alter its morphology; however, the administration of either the M9MG6-B2 or the M9MG6-B2[pRCR12] strain, with or without Incaparina, significantly increased the ratio of the villus height/crypt depth (Figure 5). These results revealed that the bacterial supplementation increased the total surface area of the intestinal villi and would thus be beneficial to nutrient absorption. This hypothesis is in line with recent results showing that the supplementation with folate-producing LAB resulted in an increase of the intestinal absorption surface (Cucick et al., 2020). Apart from the increased ratio of the villus height/crypt depth by the bacterial supplementation, no other changes were observed in the muscle layer or in the mucous layer of the small intestine.

In the fecal samples of the animals that consumed M9MG6-B2[pRCR12] in the presence or absence of Incaparina, the strains were recovered at a concentration of 4 × 10^4^ CFU mL^−1^ as determined by counting the pink colonies in MRS-agar containing CM. This result is similar to those previously obtained with strain M5MA1-B2[pRCR12], where the mCherry labeling was shown to be effective in following the survival of the strain in conventional mice (Mohedano et al., 2019). It was not possible to evaluate M9MG6-B2 recovery since this strain did not produce the mCherry protein and could not be differentiated from the normal intestinal microbiota of the mice. In addition, the presence of pRCR12 in M9MG6-B2 resulted *in vitro* in a slightly decreased growth rate (Table 4) and in an increased sensitivity of the bacterium to intestinal stress (Figure 4). Therefore, it is feasible that the *in vivo* colonization capability of M9MG6-B2 could be higher than that of M9MG6-B2[pRCR12].

Nevertheless, the overall results support a potential probiotic effect of *L. plantarum* M9MG6-B2 and its usage for the preparation of cereal-based functional food.

## Conclusions

Currently, functional foods with potential health benefits are attracting increasing interest in which food-preserving microorganisms, like probiotics, play significant roles. According to Liu et al. (2017), probiotics not only provide high levels of nutraceuticals to the food, they also participate in health regulation of humans by production of functional molecules *in situ* in the GIT. In this context, our current results revealed that both *L. plantarum* M5MA1-B2 and M9MG6-B2 are able to tolerate the GIT stresses and to grow after adaptation to these conditions. Incaparina, as a food matrix, seems to provide the studied bacteria with the adequate nutrients to allow their proliferation, therefore, the possibility of carrying them in such a matrix would assure the production of commercial preparations with a high viability of probiotic LAB with additional functionalities, for the development of new functional foods. Moreover, both the M5MA1-B2 and M9MG6-B2 strains showed *in vitro* high adhesion capacity to Caco-2 epithelial cells as well as an aggregative phenotype and capability to form consistent and high biomass biofilms, which indicate a potential capability for colonization of the intestines. Furthermore, in a murine *in vivo* model, administration of M9MG6-B2 or M9MG6-B2[pRCR12] resulted in an increment in the surface area of the intestinal epithelium (without affecting the health of the animals) that could contribute to better absorption of the nutrients.

## Data Availability Statement

All datasets generated in this study are included in the article/Supplementary Material.

## Ethics Statement

The animal study was reviewed and approved by the Animal Protection Committee of Centro de Referencia de Lactobacillus, Tucuman, Argentina.

## Author Contributions

AH-A and SP performed all the *in vitro* analysis of the LAB studied. MM planed the riboflavin production experiments, analysis of the results, and corrected the manuscript. RA provided the background for handling the riboflavin-producing LAB and corrected the manuscript. JL, GV, and AM performed the LAB analysis in the animal model, the description of the results, and corrected the manuscript. PL masterminded the direction and conceptualization of the study, the interpretation of the data obtained, and the final version of the manuscript. All authors listed have read and approved the final version of the manuscript.

## Conflict of Interest

The authors declare that the research was conducted in the absence of any commercial or financial relationships that could be construed as a potential conflict of interest.
