# Supplementary material for: The Ability of Riboflavin-Overproducing Lactiplantibacillus plantarum Strains to Survive Under Gastrointestinal Conditions
Source: Front Microbiol. 2020 Oct 22;11:591945. doi: 10.3389/fmicb.2020.591945 (PMC7649808; doi:10.3389/fmicb.2020.591945)
Supplement: Supplementary file 1 [file Data_Sheet_1.docx]

The ability of riboflavin-overproducing *Lactiplantibacillus plantarum* strains to survive under gastrointestinal conditions

Annel M. Hernández-Alcántara^1^, Sandra Pardo^1^, Mari Luz Mohedano^1^, Graciela Vignolo^2^, Alejandra de Moreno de LeBlanc ^2^, Jean Guy LeBlanc^2^, Rosa Aznar^3,4^ and Paloma López^1*^

^1^Department of Microorganisms and Plant Biotechnology, Margarita Salas Center for Biological Research (CIB-CSIC), Madrid, Spain

^2^Reference Centre for Lactobacilli (CERELA-CONICET), San Miguel de Tucumán, Tucumán, Argentina

^3^Department of Preservation and Food Safety Technologies, Institute of Agrochemistry and Food Technology (IATA-CSIC), Paterna, Spain

^4^Department of Microbiology and Ecology, University of Valencia, Valencia, Spain

**
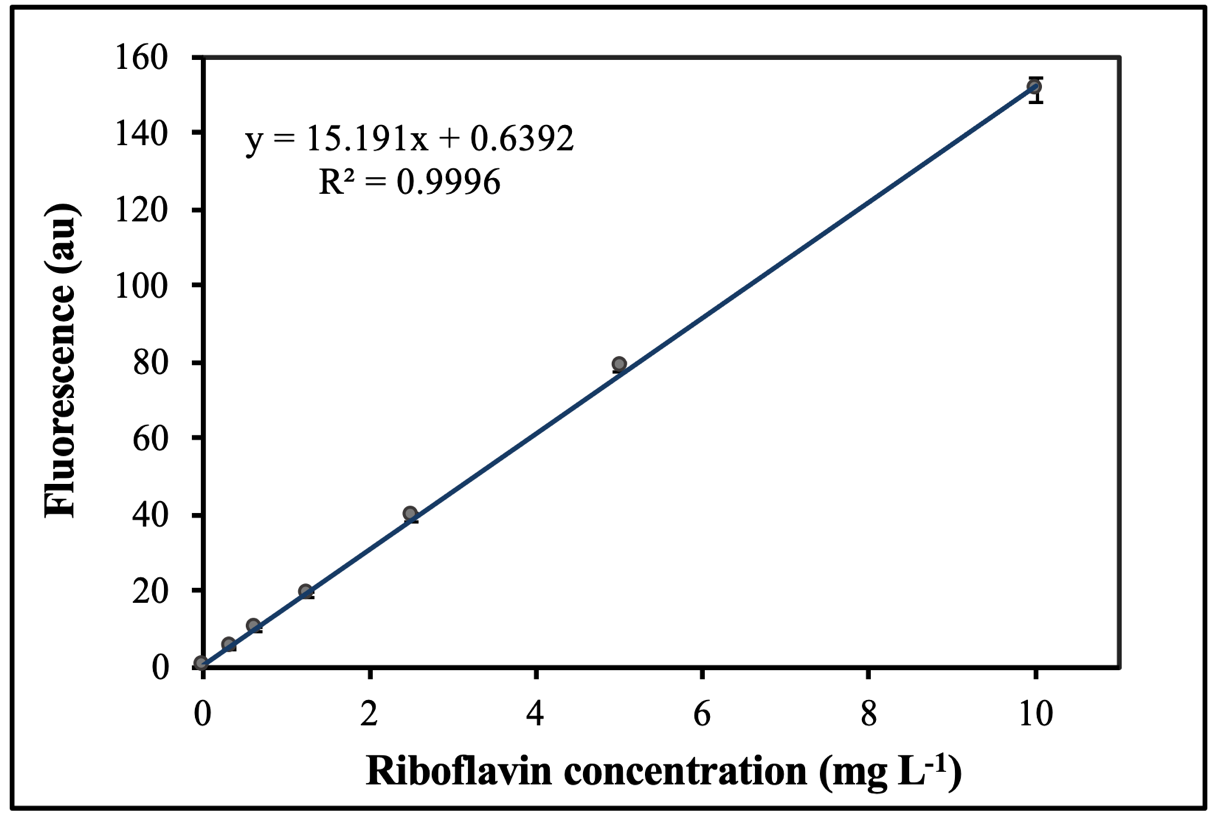
**

**Supplementary Figure S1**. **Riboflavin calibration curve.** Correlation of riboflavin concentration and fluorescence. Serial dilutions of a riboflavin solution in CDM medium lacking riboflavin at 10 mg mL^-1^ were used to determine its fluorescence emission at a wavelength of 520 nm after excitation at a wavelength of 440 nm.


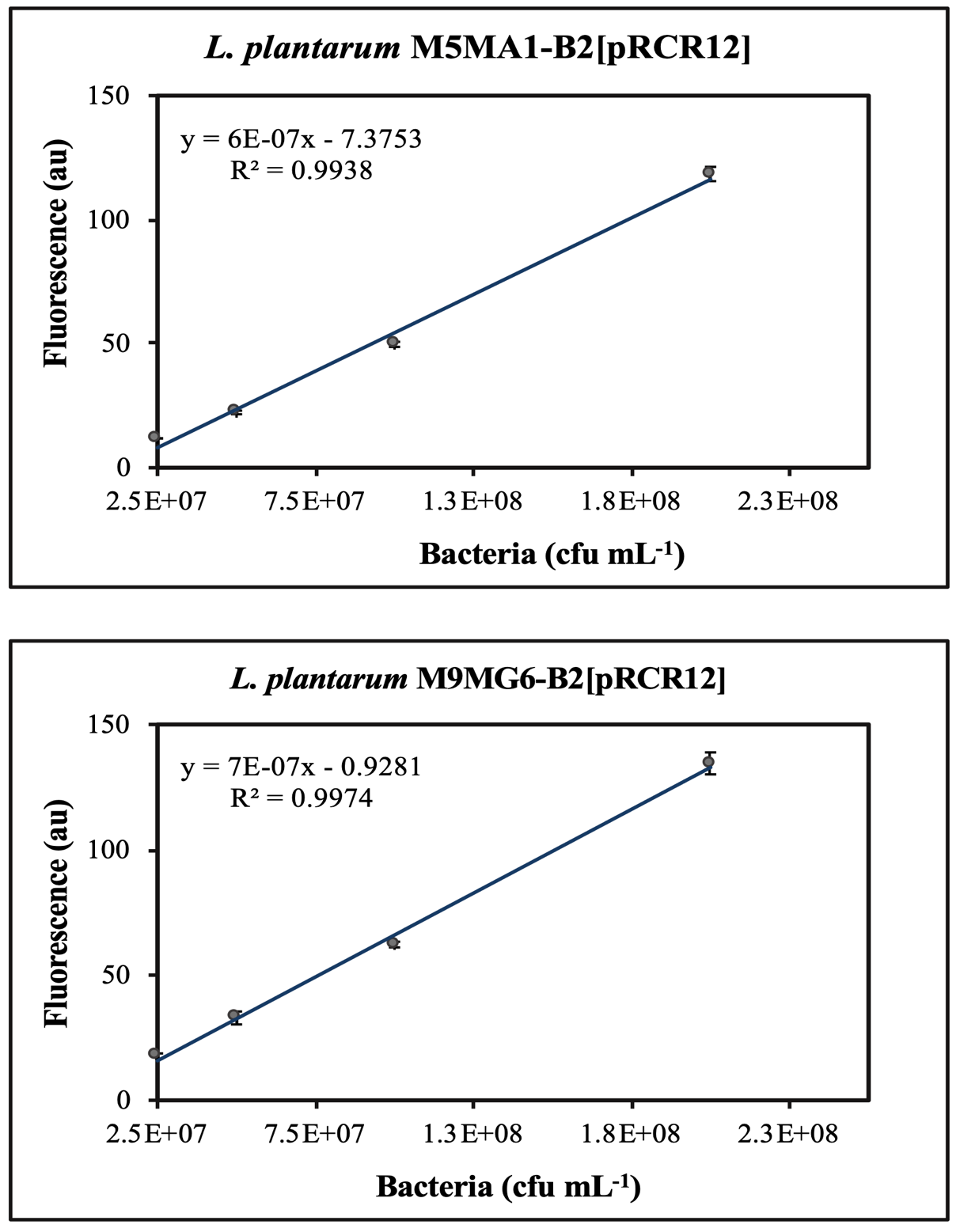


**Supplementary Figure S2. Calibration curves of *L. plantarum* M5MA1B2[pRCR12] and M9MG6-B2[pRCR12] strains**. Correlation of bacterium concentration and fluorescence due to mCherry protein were determined. Serial dilutions of a bacterial suspension at an initial concentration of 2×10^8^ cfu mL^-1^ in saline solution were employed to determine the corresponding fluorescence emission at a wavelength of 610 nm upon excitation at 587 nm. The mean values of three independent determinations and their standard deviations are depicted.

**
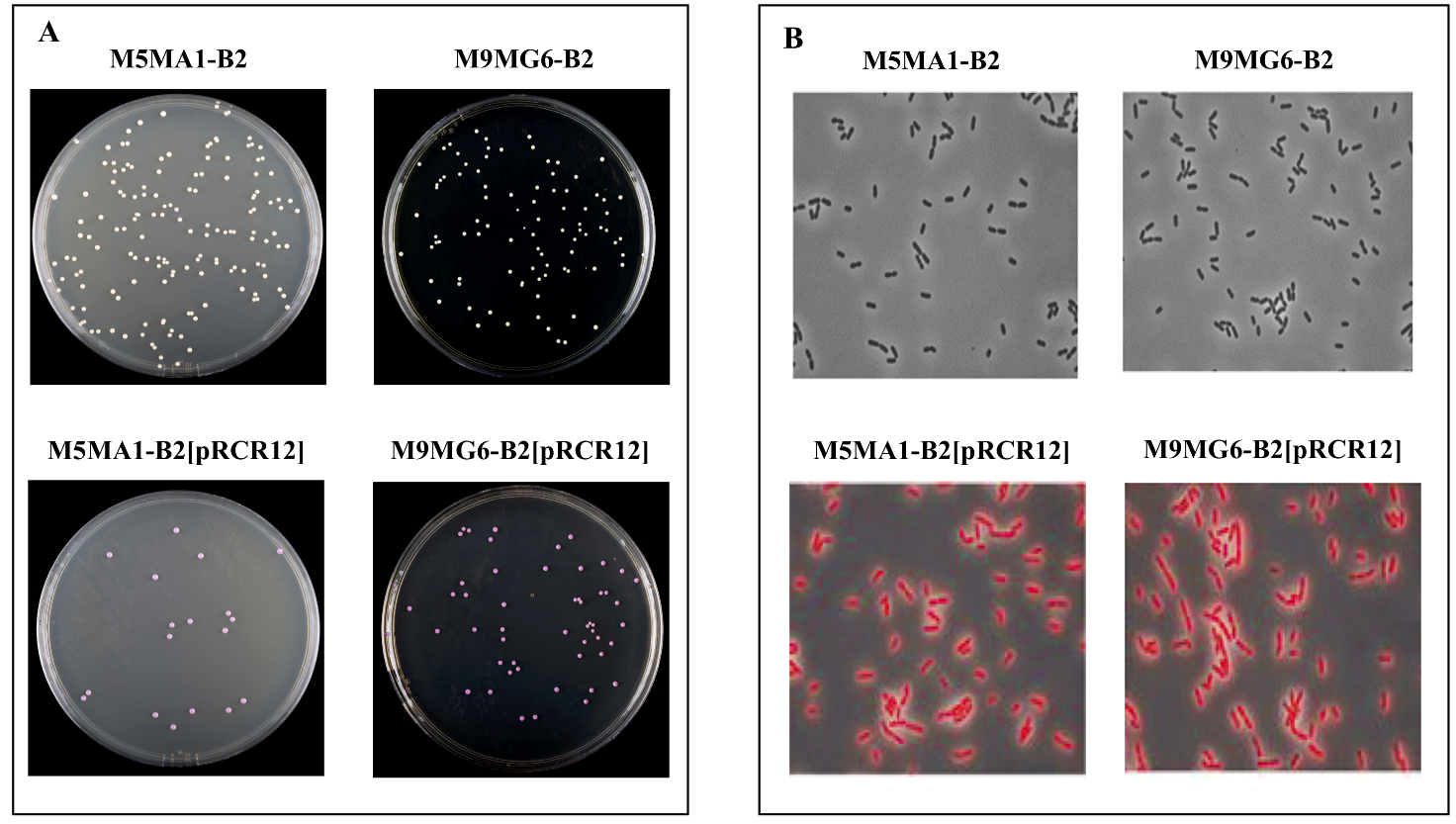
**

**Supplementary Figure S3. Analysis of the *L. plantarum* M5MA1-B2 and M9MG6-B2 strains carrying or lacking pRCR12 plasmid. (A)** Colony phenotypes of the strains is depicted. (B) Micrographs of bacterial preparations with 100 X magnification, analyzed with a Leica DM1000 model microscope and with a light source EL6000 and the filter system TX2 ET for detection of the mCherry fluorescence.

**Supplementary Table S1.** Commercial INCAPARINA composition, produced by Central de Alimentos, S.A. (Guatemala City, Guatemala).

|  |  | %of RV* |
| --- | --- | --- |
| **Energy** | **125 kcal** |  |
| Energy from fat | 25 kcal |  |
| **Total fat** | **3 g** |  |
| Saturated fat | 1.5 g |  |
| **Cholesterol** | **10 mg** |  |
| **Sodium** | **40 mg** |  |
| **Potassium** | **200 mg** |  |
| **Total carbohydrates** | **20 g** |  |
| Sugars | 12g |  |
| Dietary fibers | 1g |  |
| **Proteins** | **4g** | 8 |
| **Vitamins and ions** |  |  |
| Vitamin A | 80 μg | 10 |
| Vitamin B1 | 0.144 mg | 12 |
| Vitamin B2 | 0.216 mg | 18 |
| Vitamin B12 | 2.4 μg | 40 |
| Vitamin D | 1.5 μg | 30 |
| Folic acid | 60 μg | 15 |
| Niacin | 1.5 mg | 10 |
| Iron | 1.68 mg | 12 |
| Zinc | 1.8 mg | 12 |
| Calcium | 200 mg | 20 |

Quantities per package of Incaparina (resuspension in 200 mL).

*Reference Values (RV) according to the FAO/WHO Codex Alimentarius.
